# Knee Capsule Anatomy: An MR Imaging and Cadaveric Study

**DOI:** 10.3390/diagnostics11111965

**Published:** 2021-10-22

**Authors:** Aristeidis H. Zibis, Evangelia E. Vassalou, Vasileios A. Raoulis, Vasileios Lampridis, Michail E. Klontzas, Apostolos Fyllos, Panagiotis Stavlas, Apostolos H. Karantanas

**Affiliations:** 1 Laboratory of Anatomy, Department of Medicine, School of Health Sciences, University of Thessaly, 3 University Str, Biopolis, 41110 Larissa, Greece; ahzibis@gmail.com (A.H.Z.); v_raoulis@yahoo.gr (V.A.R.); apofyl@hotmail.com (A.F.); 2 Department of Medical Imaging, University Hospital, 71500 Heraklion, Greece; vassalou.e@hotmail.com (E.E.V.); miklontzas@gmail.com (M.E.K.); 3 Department of Medical Imaging, General Hospital of Sitia, 72300 Sitia, Greece; 4 Department of Trauma and Orthopaedics, 424 Military General Hospital, Peripheriaki Odos Efkarpias, 56429 Thessaloniki, Greece; vlampridis@hotmail.com; 5 Department of Radiology, Medical School, University of Crete, 71500 Heraklion, Greece; 6 Department of Orthopaedics, Thriasio General Hospital, 19600 Athens, Greece; stavlaspanagiotis@yahoo.gr

**Keywords:** external fixator, knee joint capsule, intra-capsular insertion, MR imaging/knee joint, MR arthrography, cadavers/knee joint

## Abstract

This research focuses on the anatomical insertion of the synovial capsule around the knee. The attachments of the capsule were measured in 50 knee MR imaging studies with large intraarticular effusion. Corresponding measurements were performed in 20 fresh frozen cadaveric specimens, for validation. Femoral and tibial capsular reflections were defined as the distances between the attachment sites of the capsule and the femoral or tibial joint line and they were recorded in three coronal planes (anterior/middle/posterior). On MR imaging, the lateral/medial femoral capsular reflection mean values were 6.5/4.57 cm, 2.74/1.74 cm and 1.52/1.99 cm in the anterior, middle and posterior plane, respectively. MR imaging-based measurements did not differ significantly compared to corresponding cadaveric measurements. The mean values of the lateral/medial tibial capsular reflection on MR imaging were 0.09/0.11 cm, 0.34/0.26 cm and 0.62/0.34 cm in the anterior, middle and posterior plane, respectively. On cadaveric dissection, the maximum mean value was 1.45 cm, measured on the lateral side of the anterior plane. Apart from the lateral aspect of the posterior plane, MR imaging measurements were significantly lower, compared to the corresponding cadaveric measurements. The greatest femoral and tibial capsular reflections were found on the anterior and lateral side of the anterior plane. MR imaging appears to underestimate the distal extent of the knee capsule. Anatomical details of the knee capsule should be considered for safe insertion of external fixator pins.

## 1. Introduction

External fixation offers advantages for the definitive treatment of complex periarticular fractures around the knee joint. Additionally, it is the method of choice for the initial stabilization of patients for damage control, when other life-threatening injuries coexist. External fixator pins in the proximal tibia are most commonly used; however, specific injuries may necessitate insertion of pins in the distal femur. 

Several complications have been associated with the application of external fixation, with pin loosening, frame failure, infection and injury of neurovascular bundles, representing the most significant [1,2,3,4,5,6]. Pin track infection has been reported as the most prevalent complication [3,7], which may be associated with septic arthritis in case of intraarticular pin insertion. In this regard, detailed knowledge of the anatomical insertion sites of the knee joint capsule on the distal femur and proximal tibia is essential in order to provide safe corridors for external fixation and ensure proper pin placement combined with low complication rate. 

The attachment sites of the knee joint capsule to the proximal tibia have been sporadically described in anatomical studies using cadavers and MR imaging data [8,9,10,11,12]. Most studies suggest that pin insertion at least 8–14 mm distal to the proximal tibial subchondral line excludes intracapsular placement [8,10,11]. On the other hand, the inter-individual variation of knee capsular insertion on the distal femur is less well described in the literature [4,6,13]. 

In the present study, we sought to define the anatomical attachment sites of the knee joint capsule to the distal femur and proximal tibia, through reviewing MR imaging examinations of knees with intraarticular effusion. Cadaveric dissection of fresh frozen knee specimens was used for validation of the MR imaging-based data. 

## 2. Materials and Methods

### 2.1. Patients

For the evaluation of the anatomical insertion sites of the knee joint capsule on the distal femur and proximal tibia, 50 knee MR imaging studies (24 left and 26 right knees) from 50 patients (33 male, 17 female; mean age, 49.6 ± 10.5 years) were prospectively evaluated. Inclusion criteria encompassed clinical signs of large intraarticular joint effusion confirmed by MR imaging. The definition and the method of semi-quantification of large intraarticular effusion on MR imaging is provided in the “MR imaging protocol and analysis” section. Joint effusions were due to traumatic hemarthrosis in all patients. Individuals with a clinical history and MR imaging findings compatible with acute or previous fracture around the knee, joint capsule rupture, articular surface collapse of any cause or severe osteoarthritis were excluded from the study.

### 2.2. MR Imaging Protocol and Analysis

All MR imaging studies were reviewed by a radiologist with seven years of experience in musculoskeletal imaging who was blind to the patient data and cadaveric dissection measurements. MR imaging protocol included fat-suppressed proton density-weighted (PD-w) or intermediate-weighted (IM-w), turbo spin echo (TSE) sequences in the axial, sagittal and coronal anatomical planes, performed on 1.5 MR imager (Vision/Sonata, Siemens, Erlangen). Image analysis was performed with the use of Evorad RIS/PACS system (Evorad SA, Athens, GR, www.Evorad.com, accessed on 10 October 2021)

**Figure 1:**
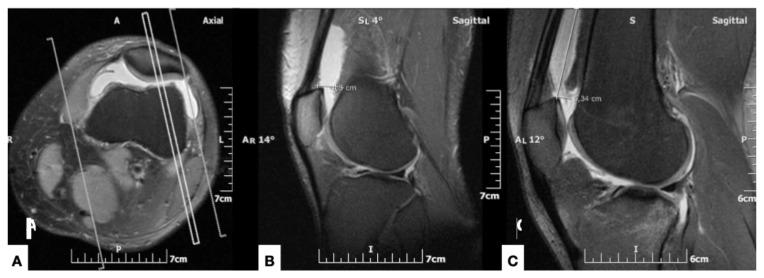
Inclusion criteria using axial (**A**) and sagittal ((**B**) and (**C**)) fat-suppressed proton density-weighted MR images. (**A**) At the level of half the patellar craniocaudal diameter, the reference for the mid-sagittal section of the patella is shown (double line). (**B**) Mid-sagittal section of the patella, shows the measurement of the anteroposterior diameter of the supra-patellar bursa, at the level of the upper pole of the patella. (**C**) At the same level as (**B**), in a different patient, the measurement of the craniocaudal dimension of the suprapatellar bursa is shown.

The intraarticular knee effusion was defined as “large” when it extended in the suprapatellar pouch for a distance of more than 5 cm proximal or more than 1 cm posterior to the proximal pole of the patella, at the mid-sagittal plane. This corresponded to an effusion volume of more than 50 mL, as validated with MR arthrographic examinations in patients who underwent an intraarticular injection of 50 mL of contrast solution (Figure 1).

**Figure 2:**
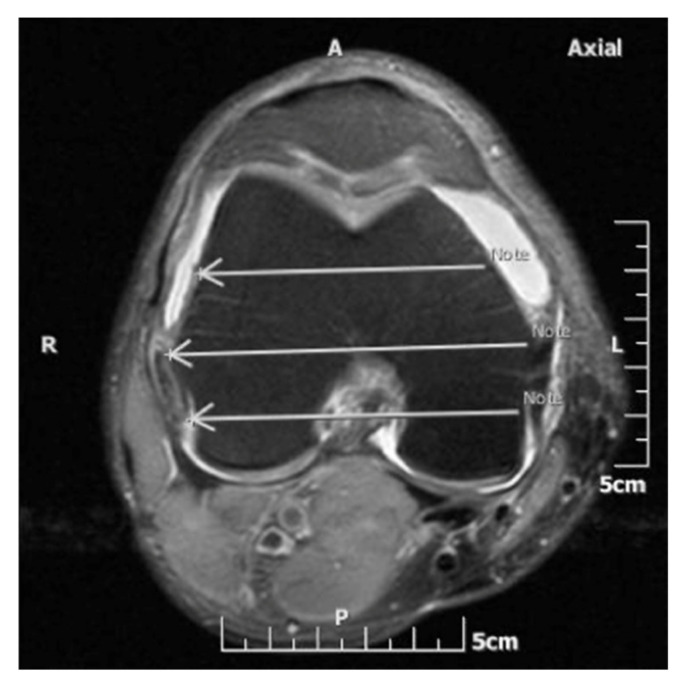
Axial fat-saturated proton density-weighted MR image at the level of the maximum mediolateral diameter of the femoral epicondyles. The middle plane (middle arrow) connects the medial and lateral femoral epicondyles. The anterior and posterior planes (anterior and posterior arrow, respectively) are drawn half way between the middle plane and a line tangent to the anterior and posterior femoral cortical margins, respectively.

**Figure 3:**
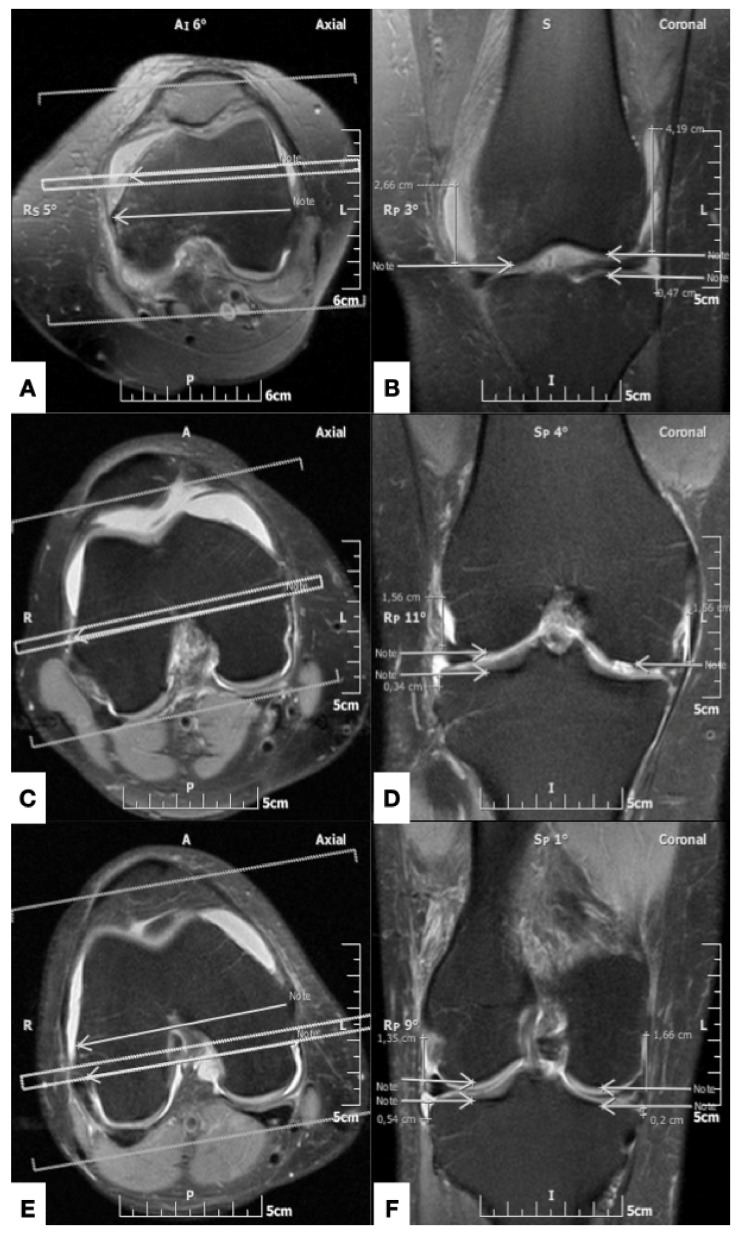
(**A**), (**C**) and (**E**), similar to Figure 2, shows the anterior, middle and posterior planes, respectively (double line in (**A**), (**C**) and (**E**), respectively), giving reference to the coronal section appearing in (**B**), (**D**) and (**F**). (**B**) Coronal fat-suppressed TSE intermediate-weighted MR image, shows the measurement of the medial femoral capsular reflection (distance between the left arrow and the superior margin of the knee intraarticular effusion), the lateral femoral capsular reflection (distance between the right top arrow and the superior margin of the effusion) and the lateral tibial capsular reflection (distance between the right bottom arrow and the inferior margin of the effusion), along the anterior plane. There is no measurable medial tibial capsular reflection on this plane. (**D**) Coronal fat-suppressed TSE intermediate-weighted MR image shows the measurements of the medial femoral capsular reflection (distance between the right arrow and the superior margin of the knee intraarticular effusion), the lateral femoral capsular reflection (distance between the left top arrow and the superior margin of the effusion) and the lateral tibial capsular reflection (distance between the left bottom arrow and the inferior margin of the effusion), in the middle plane. There is no measurable medial tibial capsular reflection on this plane. (**F**) Coronal fat-suppressed TSE intermediate-weighted MR image shows the measurements of the medial femoral capsular reflection (distance between the right top arrow and the superior margin of the knee intraarticular effusion), the lateral femoral capsular reflection (distance between the left top arrow and the superior margin of the effusion), the lateral tibial capsular reflection (distance between the left bottom arrow and the inferior margin of the effusion) and the medial tibial capsular reflection (distance between the right bottom arrow and the inferior margin of the effusion), along the middle plane.

The femoral capsular reflection was defined as the distance between the most proximal site of the intraarticular effusion and the subchondral line of the distal femur. Correspondingly, the tibial capsular reflection represented the distance between the most distal site of the joint effusion and the proximal tibial subchondral line. The femoral and tibial capsular reflections were measured in centimeters at the medial and lateral aspect of the knee joint along three coronal planes which were defined on the axial MR image at the level of femoral epicondyles: (i) the middle plane, which was drawn along a line through the femoral epicondyles, at the level of their maximum mediolateral distance on the selected axial image; (ii) the anterior plane, which passed through a line in the mid-distance between the middle plane and a line tangent to the anterior margin of the medial femoral condyle; and (iii) the posterior plane, which was drawn through a line in the mid-distance between the middle plane and a line tangent to the posterior aspect of the medial femoral condyle (Figure 2). Consequently, six measurements (three on the lateral and three on the medial aspect of the knee) for the femoral and tibial capsular reflections were recorded for each knee MR imaging examination (Figure 3).

### 2.3. Cadaveric Dissection

**Figure 4:**
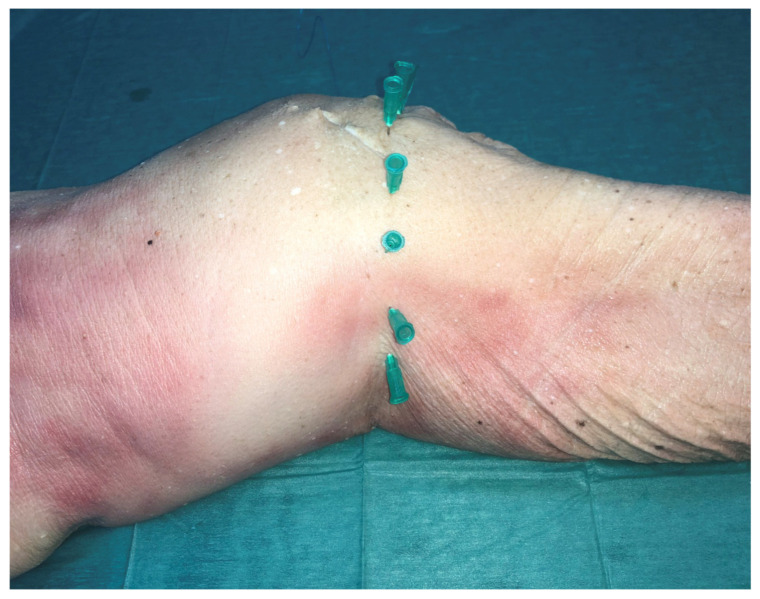
Marking the joint line with the help of pins which were placed 1 cm apart.

**Figure 5:**
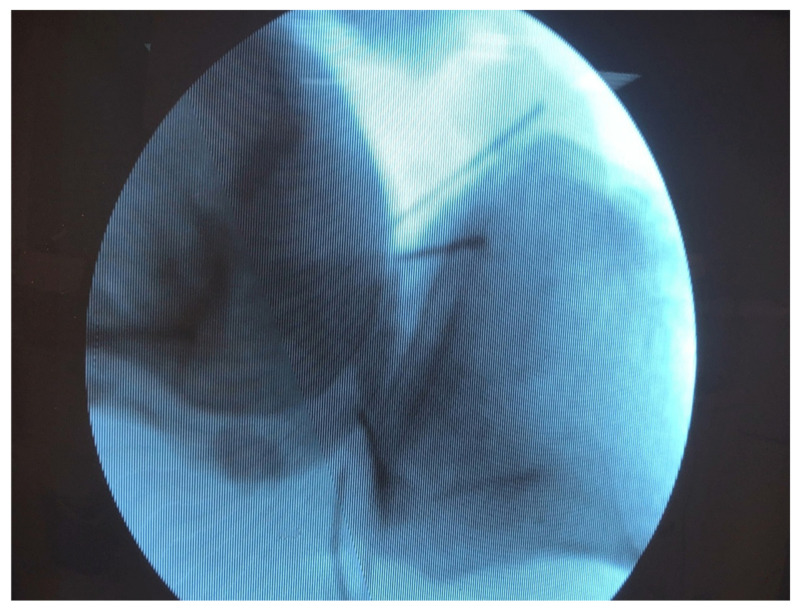
X-rays confirmed the proper placement of the pins.

**Figure 6:**
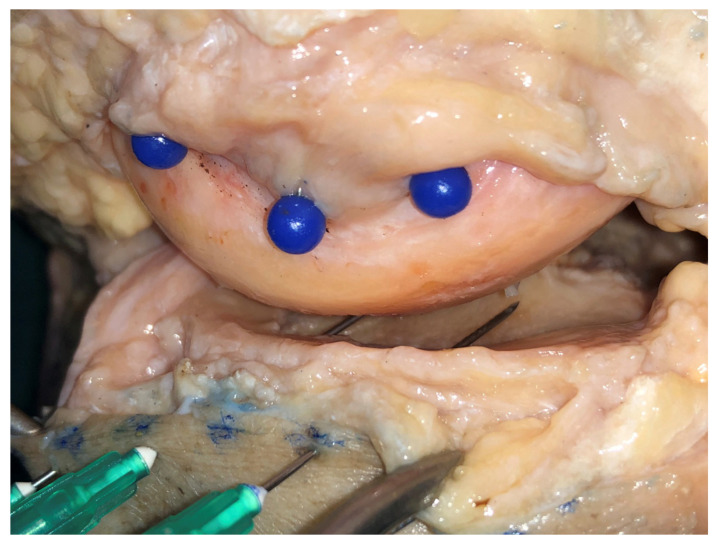
After dissection, marking the insertion of the capsule with blue pins in the femur.

In order to provide validation for the MR imaging-based measurements, cadaveric dissection was performed in a set of 20 fresh-frozen cadaveric knees (12 male, 8 female; mean age, 68.81 ± 7.0 years) which were obtained through an Anatomy Donation Program and stored at −21 °C. The specimens were thawed for 24 h before dissection at room temperature (18°). There was no medical history of any bone or soft tissue injury, surgery or osteoporosis in any of the 24 fresh frozen knee cadavers. Before starting the preparation of the cadaver knee, the joint line was marked with the help of X-rays and pins which were placed 1 cm apart (Figure 4 and Figure 5). Afterwards a skin incision was made around the knee; the incision was formed by joining the entry points of the pins. The next step was to cut carefully all extraarticular soft tissue of the knee (patella tendon, lateral collateral ligament, medial collateral ligament, vessels and nerves) and finally the capsule entering into the joint (Figure 6). Finally, in a similar manner to the MR imaging experiment, the lateral and medial condyles were divided into three equal parts/quadrants (anterior, middle and posterior) and from each of these three points the insertion of the capsule was located and the distance to the joint line was measured. All measurements were made using the same Vernie caliper (accuracy 0,01 mm).

### 2.4. Statistical Analysis

Statistical analysis was performed using GraphPad Prism version 8.4.2. Data range, mean and standard deviation were calculated. Statistical significance was accepted as *p* value < 0.05. Comparison between cadaveric and MR imaging measurements for each anatomical region was made using a 2-way ANOVA test with Sidak post-hoc analysis. 

## 3. Results

On MR imaging, the values of the femoral capsular reflections showed great inter-individual variability. The maximum mean value of the femoral capsular reflection was 6.5 cm, as assessed on the lateral side of the anterior plane. Specifically, the mean values ± standard deviation (SD) of the femoral capsular reflection on the lateral side of the knees were 6.50 ± 1.39 (range, 2.13–9.43 cm), 2.74 ± 1.66 (range, 0–8.35 cm) and 1.52 ± 0.96 (range, 0–4.16 cm) in the anterior, middle and posterior plane, respectively. On the medial aspect of the joint the corresponding values were 4.57 ± 1.60 (range, 0.98–8.16 cm), 1.74 ± 1.14 (range, 0–6.53 cm) and 1.99 ± 1.14 (range, 0–4.63 cm). MR imaging-based measurements of the femoral capsular reflection did not differ significant compared to the corresponding measurements derived from the cadaveric experiment (Table 1). 

The tibial capsular reflection values also exhibited great variability. Regarding the MR imaging-based measurements, the maximum mean value was 0.62 cm as measured on the lateral side of the posterior plane. In detail, the mean values ± SD of the tibial capsular reflection distances on the lateral aspect of the knees were 0.09 ± 0.15 (range, 0–0.51 cm), 0.34± 0.19 (range, 0–0.78 cm) and 0.62 ± 0.53 (range, 0–1.9 cm) in the anterior, middle and posterior plane, respectively. On the medial side, the corresponding measurements were 0.11 ± 0.23 (range, 0–1.03 cm), 0.26 ± 0.37 (range, 0–1.49 cm) and 0.34 ± 0.58 (range, 0–2.03 cm). MR imaging measurements of the tibial capsular reflection were significantly lower at all sites, apart from the lateral aspect of the posterior plane, compared to the corresponding measurements obtained from the cadaveric specimens. Regarding the cadaveric dissection, the maximum mean value of the tibial capsular reflection was 1.45 cm, as measured on the lateral side of the anterior plane (Table 2). This section may be divided by subheadings. It should provide a concise and precise description of the experimental results, their interpretation, as well as the experimental conclusions that can be drawn.

## 4. Discussion

External fixators that require insertion of pins into the distal femur and proximal tibia are indicated in trauma for initial stabilization or definitive treatment, as well as in elective deformity corrections. Knowledge of the anatomy of the knee capsule is essential to avoid intra-capsular placement of pins, and the potentially associated complications such as septic arthritis. Although the incidence of septic arthritis related to intracapsular penetration was found to be low in one prospective study (1 out of 145 patients, <0.7%), it is a serious complication which can be obsolete provided that normal anatomy is taken into consideration [4]. The knee surgeon, apart from the joint capsule topography, should be up to date with common anatomical variations in the course of the saphenous nerve and important vascular structures that are closely related to the joint capsule [14,15]. However, the exploration and documentation of anatomical structures outside the knee joint capsule was not the scope of the present study.

There are few recommendations on the safety zone for pin placement in the distal femur. In a cadaveric study conducted by McElvany et al., 29% of external fixator pins that were placed at the level of the adductor tubercle were found to have penetrated the knee capsule [6]. In the same study, pins that were leveled with the superior pole of the patella were also associated with intracapsular placement in 7% of the specimens [6]. Authors concluded that pins should be inserted at least 0.7 cm proximal to the adductor tubercle. Furthermore, Lowery et al. demonstrated that on the medial side, the capsule reflection can be up to 74% of the distance between the anterior aspect of the medial femoral condyle and the adductor tubercle [13]. Authors of this study also recommend pin placement proximal to the adductor tubercle. The capsule reflection on the lateral side was also found to attach more posteriorly, by up to 57% of the anterior-posterior femoral diameter [13]. The authors therefore suggested that pins should exit more posteriorly to avoid penetration of the capsule. The mean distance between the adductor tubercle and the knee joint line has been suggested as 44 ± 4.27 mm [16]. Herein, we found that the femoral capsular reflections in the anterior plane, with a mean value of 6.5 cm and 4.57 cm for the lateral and medial side, respectively, were larger compared to other studies. The values of the femoral capsular reflections at the other sites were comparable to previous reports. 

The measurements of the capsular attachments to the proximal tibia are better described in the literature. Stevens et al. conducted a cadaveric study to determine the cause of pin placement associated septic arthritis [11]. Authors concluded that the mean distance from the tibial joint line was 8 mm posteromedially, 3 mm anteriorly and 11 mm posterolaterally and suggested that distances greater than the reported values are recommended for safe insertion of pins. DeCoster et al. measured the capsule insertion on the proximal tibia in one anterior and three posterior regions and found that the mean distance from the joint line was 6 mm anteriorly and 12 mm posteromedially and posterolaterally [8]. The authors suggested that the safety zone is 14 mm from the subchondral line. Reid et al. performed measurements of distances from the subchondral line to the capsular attachment in three medial quadrants and two lateral quadrants on MRI images [10]. They also recommended that the safety zone of pin placement is 14 mm distal to the subchondral line [10,17]. In contrast to the other studies, Hyman and Moore demonstrated greater distances (0–70 mm) of the capsular reflection on the proximal tibia, with significant inter-individual variations [9]. In this study, a higher incidence of tibiofibular joints connecting with the knee joint was also found (50%) compared with that found in other studies (10%) [8,9,10]. In the present study, we found significant inter-individual variation regarding the tibial capsular reflection values. The mean capsular attachment distances on the proximal tibia, as assessed with cadaveric dissections, were similar to that found in other studies, and it concurs with the recommendation of an insertion point at least 14 mm distal to the tibial joint line. 

Significant differences between the cadaveric and MR imaging measurements regarding the definition of the tibial capsular reflection were also found, with imaging data underestimating the distal extension of the knee capsule. This may be attributed to the short distance between the insertion site of the capsule and the joint line which was measured in a sub-centimeter scale inducing potential challenges in accurate measurement when using MR imaging. Additionally, considering the high variability in the average knee joint volume capacity among individuals, being reported to range between 40–290 mL [14], the defined large intraarticular effusions, according to our methods, may not suffice to ensure capsular distention in all cases. 

Our study has certain strengths and limitations. The number of the recorded MR imaging-based measurements for the determination of the femoral and tibial capsular reflection, being the largest in the reported literature, represents a strength of the present study. Additionally, the conduction of the cadaveric experiment provided validation of the imaging data. This is of importance as it enhances the value of MR imaging in defining the attachment sites of the knee capsule, considering that there were not significant differences between the two methods, at least regarding the evaluation of the femoral capsular reflections. Our study has specific limitations. Firstly, apart from the cadaver donors’ age and medical history, no other information was available that could reflect on the tissues quality and therefore might influence the measurements. The body mass index was also not known (or recorder) for both the cadaveric and the MR imaging group. Furthermore, the cadaveric and MR imaging groups were not age and gender matched. However, previous studies did not find the average volume of the knee joint to be age- or sex-dependent [18]. Secondly, non-assessment of inter- and intra-rated agreement for the MR imaging measurements may be regarded as a limitation. However, the manual process of the capsular reflection measurements involved the identification of well-defined bone and soft-tissue structures, thus eliminating potential bias. Finally, the semi-quantification of the knee joint effusions on MR imaging, may not ensure capsular distention in all cases, considering the high variability in the average knee joint volume among individuals.

## 5. Conclusions

The greatest femoral capsular reflections were found on the anterior plane and exceeded previously reported values. On MR imaging the greatest tibial reflections were found laterally on the posterior plane; however, the method appears to underestimate the distal extent of the knee capsule, considering cadaveric dissection as the reference standard. Cadaveric dissections revealed the maximum tibial capsular reflections to be located on the lateral aspect of the anterior plane with the assessed values being consistent with those of previous studies and existing guidance. Detailed understanding of the knee joint anatomy is essential for determining safe corridors in order to ensure uncomplicated insertion of external fixator pins. 

## Figures and Tables

**Table 1:** Mean values ± standard deviation (SD) of the measurements of the femoral capsular reflections in cadaveric specimens and MR imaging studies (ns = not significant).

Anatomical Plane	Cadaver (*n* = 20) Mean ± SD	MR Imaging (*n* = 50) Mean ± SD	Univariate Hazard Ratio (95% CI)	*p* Value
**Lateral**				
Anterior	6.47 ± 1.14	6.50 ± 1.39	(−0.87 to 0.82)	ns
Middle	2.92 ± 1.42	2.74 ± 1.66	(−0.66 to 1.03)	ns
Posterior	2.07 ± 1.03	1.52 ± 0.96	(−0.28 to 1.40)	ns
**Medial**				
Anterior	5.21 ±1.60	4.57 ± 1.60	−0.19 to 1.46	ns
Middle	1.62 ± 1.00	1.74 ± 1.14	−0.94 to 0.70	ns
Posterior	2.08 ± 1.01	1.99 ± 1.14	−0.73 to 0.91	ns

**Table 2:** Mean values ± standard deviation (SD) of the measurements of the tibial capsular reflections in cadaveric specimens and MR imaging studies (ns = not significant).

Anatomical plane	Cadaver (*n* = 20) Mean ± SD	MR Imaging (*n* = 50) Mean ± SD	Univariate Hazard Ratio (95% CI)	*p* Value
**Lateral**				
Anterior	1.45 ± 2.40	0.09 ± 0.15	(0.85 to 1.86)	<0.0001
Middle	0.91 ± 0.25	0.34 ± 0.19	(0.06 to 1.09)	<0.05
Posterior	0.96 ± 0.24	0.62 ± 0.53	(−0.17 to 0.83)	ns
**Medial**				
Anterior	0.92 ± 0.25	0.11 ± 0.23	(0.57 to 1.06)	<0.0001
Middle	0.94 ± 0.25	0.26 ± 0.37	(0.43 to 0.92)	<0.0001
Posterior	0.98 ± 0.23	0.34 ± 0.58	(0.40 to 0.89)	<0.0001
